# Impact of pre-transplant body mass index on outcomes in AML patients aged ≥ 50 years after allogeneic hematopoietic cell transplantation

**DOI:** 10.3389/fimmu.2025.1586523

**Published:** 2025-07-04

**Authors:** Wenwen Guo, Haixiao Zhang, Hongye Gao, Yawei Zheng, Mingyang Wang, Wenbin Cao, Rongli Zhang, Qiaoling Ma, Yi He, Weihua Zhai, Donglin Yang, Aiming Pang, Sizhou Feng, Mingzhe Han, Yigeng Cao, Erlie Jiang

**Affiliations:** ^1^ State Key Laboratory of Experimental Hematology, National Clinical Research Center for Blood Diseases, Institute of Hematology & Blood Diseases Hospital, Chinese Academy of Medical Sciences & Peking Union Medical College, Tianjin, China; ^2^ Haihe Laboratory of Cell Ecosystem, Institute of Hematology & Blood Diseases Hospital, Chinese Academy of Medical Sciences & Peking Union Medical College, Tianjin, China; ^3^ Tianjin Institutes of Health Science, Tianjin, China

**Keywords:** allogeneic hematopoietic cell transplantation, elderly, acute myeloid leukemia, body mass index, survival

## Abstract

**Introduction:**

The prognostic significance of body mass index (BMI) in elderly acute myeloid leukemia (AML) patients undergoing allogeneic hematopoietic cell transplantation (allo-HCT) remains controversial.

**Methods:**

This retrospective study analyzed 142 AML patients aged ≥50 years receiving allo-HCT (2013-2022), stratified by Chinese BMI criteria: low BMI (<24 kg/m², n = 83) vs. high BMI (≥24 kg/m², n = 59).

**Results:**

The median pre-transplant BMI was 23.63 (IQR, 22.07-25.78) kg/m². Multivariate analysis identified BMI <24 kg/m² as an independent risk factor for inferior OS (HR=1.80, p=0.037) and GRFS (HR=2.00, p = 0.003). Although BMI did not correlate with relapse, long-term non-relapse mortality (NRM), or the incidence of acute and chronic graft versus host disease (GVHD), the one-year NRM was significantly higher in the low BMI group compared to the high BMI group (p = 0.006). Subgroup analysis revealed that high-risk patients [not complete remission (NR) or CR but minimal residual disease (MRD)-positive) with low BMI had markedly reduced 3-year OS (20.87% vs. 57.69%, p=0.006), whereas no difference was observed in low-risk (CR/MRD-negative) patients.

**Discussion:**

Pre-transplant BMI independently predicts inferior survival in older adults with AML undergoing allo-HCT. These findings highlight the need for BMI-guided nutritional interventions, especially for high-risk older patients.

## 1 Introduction

Acute myeloid leukemia (AML) is a prevalent malignant myeloid tumor, predominantly occurring in elderly patients. Allogeneic hematopoietic cell transplantation (allo-HCT) remains the only curative treatment for high-risk AML. In recent years, advancements in transplantation technology and increased graft availability have removed age as a contraindication for allo-HCT in elderly AML patients (1, 2).

Nutritional status significantly influences morbidity and mortality in cancer patients (3). Overweight and obesity are recognized health risk factors associated with cardiovascular and metabolic comorbidities (4). For patients undergoing HCT, a pre-transplant BMI of ≥35 kg/m² has been incorporated into the hematopoietic cell transplantation-specific comorbidity index (HCT-CI) to predict post-transplant outcomes (5). However, the impact of BMI on prognosis after allo-HCT remains controversial. A study analyzing 310 adults with acute leukemia from a Chinese center found that a BMI ≥23 kg/m² improved overall survival (OS) following allo-HCT (6). Conversely, weight loss during chemotherapy was associated with inferior OS in AML patients after allo-HCT (3). Recently, a study reported that BMI at transplantation did not influence OS or non-relapse mortality (NRM) in myelofibrosis (MF) patients after allo-HCT, although its impact on relapse incidence was modestly significant (7). The role of BMI in predicting outcomes for elderly patients with AML after allo-HCT is not well established. In AML patients aged ≥60 years undergoing intensive induction chemotherapy, BMI ≥30 kg/m² emerged as an independent predictor of mortality, primarily due to obesity-related comorbidities (8). In contrast, in elderly (≥60 years) patients with myeloid malignancies undergoing allo-HCT, obesity did not impact clinical outcomes, including OS, progression-free survival (PFS), and graft-versus-host disease (GVHD) (9). Therefore, we conducted a retrospective analysis to assess the prognostic significance of BMI in elderly patients following allo-HCT.

## 2 Methods

### 2.1 Patients

This was a retrospective study including patients diagnosed with AML under the 2008 WHO guidelines who underwent their first allogeneic hematopoietic cell transplantation (allo-HCT) at age 50 years or older from January 2013 to December 2022 in our center. The stratification risk of AML was classified according to the 2022 European Leukemia Net (ELN) recommendations (10).

All patients in this study received modified myeloablative conditioning (MAC) regimens. The donors were limited to HLA-matched siblings (MSDs) or haploidentical related donors (HIDs). In total,142 patients were analyzed in this study. Patients were regularly followed up until December 2023. This research was approved by the Medical Ethics Committee of the Institute of Hematology & Blood Diseases Hospital and followed the Declaration of Helsinki.

### 2.2 Treatments

The MAC regimens consisted of intravenous busulfan (3.2 mg/kg/day for 3 days) combined with cyclophosphamide (40 mg/kg/day for 2 days). Anti-thymocyte globulin (ATG) was administered to patients for *in vivo* T-cell depletion. Calcineurin inhibitors (CNIs) with short-course methotrexate (MTX) were used for graft versus host disease (GVHD) prophylaxis. Comorbidities were assessed with the hematopoietic cell transplantation-specific comorbidity index (HCT-CI).

MRD was assessed by multiparametric flow cytometry (MFC) based on leukemia-associated immunophenotypes or by real-time quantitative PCR (qPCR) based on leukemia-associated fusion genes, including RUNX1/RUNX1T1, CBFβ-MYH11, and MLL.

### 2.3 Definitions and objectives

The primary endpoint was overall survival (OS). Relapse was defined as the presence of > 5% bone marrow blasts, blasts in peripheral blood, or extramedullary disease. Non-relapse mortality (NRM) was defined as death from any cause, except for relapse. Acute GVHD (aGVHD) was graded according to the Mount Sinai Acute GVHD International Consortium (MAGIC) criteria, and chronic GVHD (cGVHD) was classified as limited or extensive (11). GRFS was defined as survival without grade III–IV aGVHD, extensive cGVHD, or relapse. We defined MRD-positivity as an MRD ≥ 0.01% measured by MFC or MRD ≥ 0.001% measured by qPCR (12, 13).

In accordance with the recommendations of the Working Group on Obesity in China regarding weight classifications for the Chinese population, the suggested BMI categories are as follows: < 18.5 kg/m² (underweight); 18.5-23.9 kg/m² (normal-weight); 24.0-27.9 kg/m² (overweight); ≥ 28.0 kg/m² (obese) (14).Besides, we plotted the hazard ratio (HR) of OS against BMI and observed that the HR for OS approached 1 at a BMI of approximately 23.92 kg/m^2^ in this study. Therefore, we used a BMI of 24 kg/m^2^ as the cutoff to define high and low BMI groups, as illustrated in **Supplementary Figure 1**.

### 2.4 Statistical analysis

The rates of OS and GRFS were calculated using the Kaplan–Meier method. The probabilities of CIR, NRM, acute GVHD (aGVHD), and chronic GVHD (cGVHD) were calculated by competing risk analysis, accounting for competing risks. For GVHD-related NRM, relapse and NRM from other causes were defined as competing risks. Categorical variables were compared by the chi-squared test or Fisher’s exact test. The Mann–Whitney U test was used to compare continuous variables. Gray’s test and the log-rank test were used to compare variables between groups. Cox proportional hazard models were used for multivariate analysis of OS and GRFS. Fine-Gray methods were used for multivariate analysis of CIR and NRM. BMI at transplantation was included in the multivariate analysis. Besides, variables with P < 0.2 in the univariate analysis were included in the multivariate analysis. Statistical analyses were performed using R software, version 4.1.3, SPSS 20, and Graphpad Prism 10. All p values were two-tailed, and p < 0.05 was considered statistically significant.

## 3 Results

### 3.1 Patient characteristics

There were 142 AML patients aged 50 years or older who received MAC allo-HCT included in the analysis. The characteristics of the patients stratified by BMI groups are listed in **Table 1**. Among the 142 patients, 59 (41.5%) had a pre-transplant BMI of 24 kg/m² or higher. The remaining 83 patients (58.5%) had a BMI lower than 24 kg/m², of whom 3 (2.11%) had a BMI lower than 18.5 kg/m². The median pre-transplant BMI of patients was 23.63 (IQR, 22.07-25.78) kg/m². The frequency distribution of pre-transplant BMI values was illustrated in **Supplementary Figure 2**. The median follow-up time was 42.87 (95% CI, 32.82-52.92) months for patients in the low BMI group and 44.03 (95% CI, 37.73-50.33) months for those in the high BMI group (p = 0.864). A higher proportion of patients in the low BMI group did not achieve complete remission (CR) before transplantation, although the difference did not reach statistical significance (18.1% vs 8.5%; p = 0.108). Additionally, patients in the low BMI group exhibited a higher proportion of secondary AML (24.1% vs 13.6%; p = 0.120). There was no difference in other factors, including age, gender, HCT-CI scores, transplantation type, risk stratification, and GVHD prophylaxis.

**Table 1:** Patient characteristics according to pretransplant BMI.

Variables	High-BMI (≥ 24 kg/m^2^)	Low-BMI (< 24 kg/m^2^)	p
Numbers	59	83	
Median Age (IQR)	52 (51, 55)	53 (51, 55)	0.194
Age, n (%)			0.646
50-55	44 (74.6)	59 (71.1)	
≥ 55	15 (25.4)	24 (28.9)	
Gender, n (%)			0.363
male	33 (55.9)	40 (48.2)	
female	26 (44.1)	43 (51.8)	
ELN genetic risk, n (%)			0.237
favorable	18 (30.5)	16 (19.3)	
intermediate	20 (33.9)	28 (33.7)	
high	21 (35.6)	39 (47.0)	
Disease origin, n (%)			0.120
* de novo*	51 (86.4)	63 (75.9)	
secondary	8 (13.6)	20 (24.1)	
Median chemotherapy courses (IQR)	4 (3,7)	4 (3,5)	0.160
Median interval from diagnosis to transplantation, months (IQR)	7.9 (6.3, 16.7)	7.8 (6.0, 10.8)	0.236
Number of courses required to achieve CR1, n (%)			0.239
1	39 (66.1)	44 (53.0)	
≥ 2	16 (27.1)	28 (33.7)	
not remission	4 (6.8)	11 (13.3)	
Disease status, n (%)			0.108
CR and MRD negative	39 (66.1)	56 (67.5)	
CR but MRD positive	15 (25.4)	12 (14.5)	
NR	5 (8.5)	15 (18.1)	
Donor gender, n (%)			0.527
male	39 (66.1)	59 (71.1)	
female	20 (33.9)	24 (28.9)	
Donor age, n (%)			0.212
< 50	48 (81.4)	60 (72.3)	
≥ 50	11 (18.6)	23 (27.7)	
HCT-CI score, n (%)			0.148
0-1	54 (91.5)	69 (83.1)	
≥ 2	5 (8.5)	14 (16.9)	
Transplant type, n (%)			0.363
MSDT	26 (44.1)	43 (51.8)	
HIDT	33 (55.9)	40 (48.2)	
GVHD prophy, n (%)			0.611
CNI+MTX	6 (10.2)	10 (12.0)	
CNI+MTX+ATG	21 (35.6)	35 (42.2)	
CNI+MTX+MMF+ATG	32 (54.2)	38 (45.8)	
Mononucleated cell count (IQR, 10^8^/kg)	10.5 [9.0, 12.7]	10.8 [9.2, 12.4]	0.846
CD34+ cell count (IQR, 10^6^/kg)	2.9 [2.3, 3.7]	2.6 [2.3, 3.8]	0.500
Median time of neutrophil engraftment, days	13 [12, 15]	13 [11, 15]	0.594
Median time of platelet engraftment, days	14 [12, 18]	14 [12, 22]	0.908
Post-HCT prophylactic therapy, n (%)			0.333
Received	13(22.0)	13 (15.7)	
Not received	46 (78.0)	70 (84.3)	
Gender mismatch, n (%)			0.323
match	30 (50.8)	34 (41.0)	
male to female	17 (28.8)	34 (41.0)	
female to male	12 (20.3)	15 (18.1)	
Grafts source			1.000
PB	58 (98.3)	81 (97.6)	
PB+BM	1 (1.7)	2 (2.4)	

BMI, body mass index; ELN, European Leukemia-Net; CR, complete remission; MRD, minimal residual disease; NR, not completer remission; HCT-CI, hematopoietic cell transplantation-specific comorbidity index; MSDT, HLA-matched sibling transplantation; HIDT, haploidentical donor transplantation; CNI, calcineurin inhibitor; MTX, methotrexate; ATG, anti-thymocyte globulin; MMF, mycophenolate mofetil; PB, peripheral blood; BM, bone marrow.

### 3.2 Overall survival

**Figure 1:**
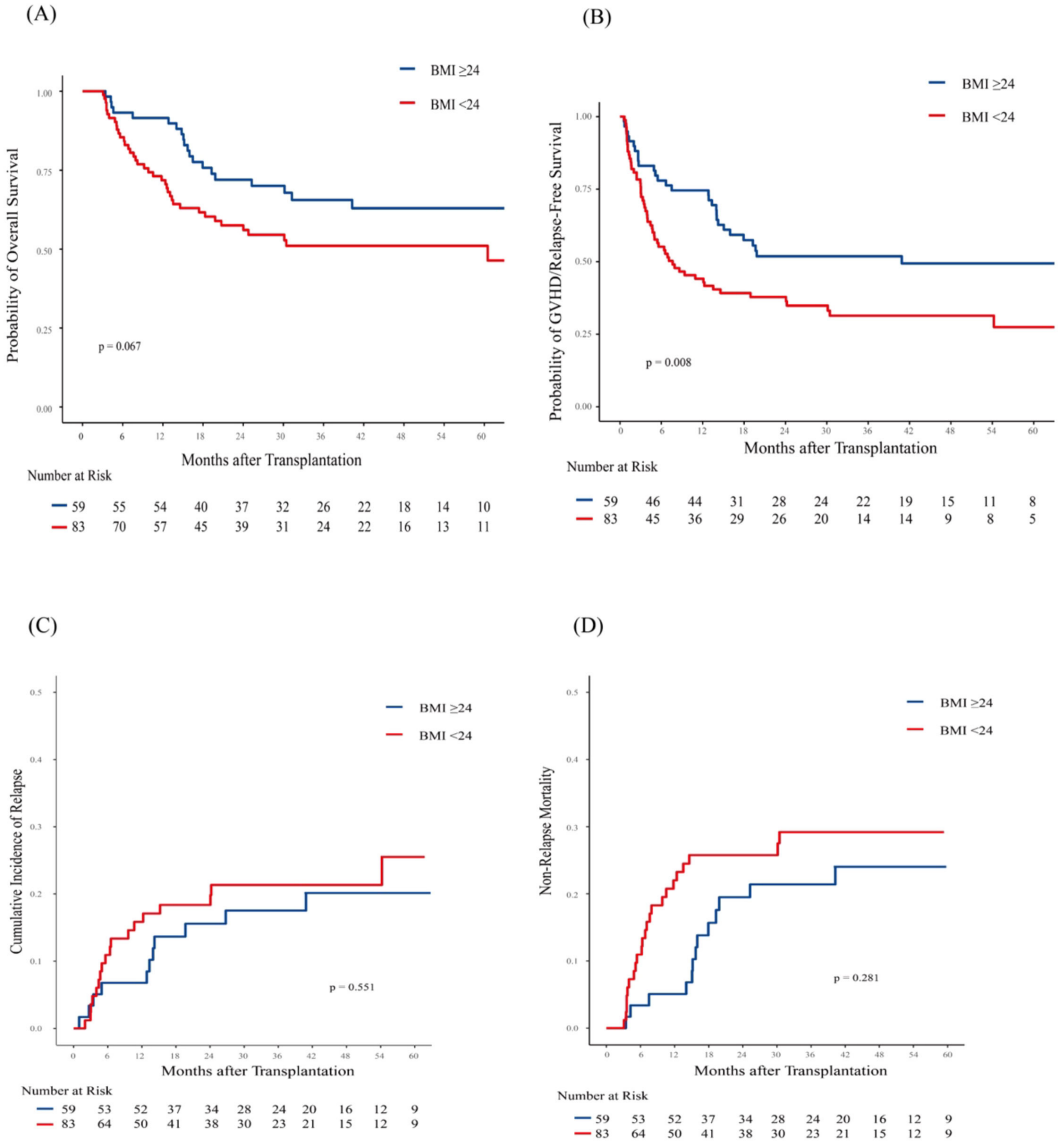
Association of body mass index (BMI) with Post-Transplantation Outcomes in 142 AML patients. Shown are **(A)** Overall survival (OS); **(B)** GVHD-free/relapse-free survival (GRFS); **(C)** Cumulative incidence of Relapse (CIR); **(D)** Non-relapse mortality (NRM). Patients with low BMI (< 24 kg/m²) exhibited significantly worse GRFS (p = 0.008). OS also showed a trend toward inferiority with low BMI, although this association did not reach statistical significance (p=0.067). No statistically significant associations were observed for CIR (p = 0.551) and NRM (p = 0.281).

During the follow-up period, 21(21/59, 35.6%) patients in the high BMI group and 39 (47.0%, 39/83) in the low BMI group died. The causes of death are detailed in **Supplementary Table 1**. GVHD was the cause of death for 8/39 (20.5%) low-BMI patients and 2/21 (9.5%) high-BMI patients. The 3-year OS was 57.10% (95% CI, 49.12%-66.36%) in the entire cohort. The 3-year OS were 65.56% (95% CI, 54.00%-79.60%) for patients in the high BMI group and 51.02% (95% CI, 40.81%-63.79%) for those in the low BMI group (p = 0.067; **Figure 1A**). Besides, patients who achieved CR and were MRD-negative had a 3-year OS of 66.51% (95% CI, 57.28%-77.24%), compared to 39.32% (95% CI, 23.91%-64.66%) for those who were CR but MRD-positive, and 35.29% (95% CI, 18.86%-66.06%) for those who did not achieve CR (NR). (p = 0.013 for CR/MRD-positive patients vs. CR/MRD-negative patients; p = 0.002 for NR vs. CR/MRD-negative patients; **Supplementary Figure 3**).

In multivariate analysis, pre-transplant BMI was found to have a significant effect on OS (HR, 1.80, 95%CI, 1.04-3.12; p = 0.037). Besides, CR but MRD positive (HR,2.92; 95%CI, 1.50-5.66; p = 0.002) and NR (HR, 2.69, 95% CI, 1.22-5.92; p = 0.014) at allo-HCT were identified as risk factors for inferior OS as well (**Table 2**).

**Table 2:** Multivariate analysis for outcomes.

Variables	OS		GRFS		CIR		NRM	
	HR (95% CI)	P	HR (95% CI)	P	HR (95% CI)	P	HR (95% CI)	P
ELN genetic risk								
favorable	1 (reference)				1 (reference)			
intermediate	1.74 (0.82-3.67)	0.147			14.02 (1.76-111.68)	0.013		
adverse	1.19 (0.58-2.43)	0.642			11.80 (1.69-82.33)	0.013		
Disease status								
CR and negative MRD	1 (reference)		1 (reference)		1 (reference)			
CR but positive MRD	2.92 (1.50-5.66)	0.002	2.35 (1.41-3.94)	0.001	6.06 (2.14-17.12)	< 0.001		
NR	2.69 (1.22-5.92)	0.014	1.92 (1.03-3.59)	0.042	2.49 (0.63-9.84)	0.19		
Pretransplant BMI (kg/m2)								
≥ 24	1 (reference)		1 (reference)		1 (reference)		1 (reference)	
< 24	1.80 (1.04-3.12)	0.037	2.00 (1.26-3.17)	0.003	1.32 (0.62-2.82)	0.470	1.44 (0.72-2.88)	0.300
Disease origin								
* de novo*	1 (reference)				1 (reference)			
secondary	1.55 (0.76-3.15)	0.226			1.34 (0.42-4.30)	0.620		
Transplantation type								
HIDT			1 (reference)					
MSDT			1.53 (0.99-2.36)	0.055				
Gender mismatch								
match							1 (reference)	
female to male							0.42 (0.14-1.27)	0.120
male to female							0.79 (0.38-1.66)	0.530
Post-HCT prophylactic therapy								
Not received	1 (reference)		1 (reference)				1 (reference)	
Received	0.45 (0.21-0.99)	0.047	0.55 (0.29-1.03)	0.063			0.47 (0.17-1.29)	0.150

ELN, European Leukemia-Net; CR, complete remission; MRD, minimal residual disease; NR, not completer remission; BMI, body mass index; MSDT, HLA-matched sibling; HCT, hematopoietic cell transplantation.

### 3.3 Graft versus host disease-free/relapse-free survival, relapse and nonrelapse mortality

Patients in the low BMI group had a significantly lower 3-year GRFS rate (31.38%, 95% CI, 22.46%-43.84%) compared to those in the high BMI group (51.86%, 95% CI, 40.43%-66.53%; p = 0.008; **Figure 1B**). In the multivariate analysis, pre-transplant BMI was an independent risk factor for inferior GRFS (HR,2.00, 95%CI, 1.26-3.17; p = 0.003).

At 3 years, the CIR was similar between patients in the high BMI group (17.54%, 95% CI, 8.92%-28.55%) and those in the low BMI group (21.34%, 95% CI, 13.03%-31.02%; p = 0.551; **Figure 1C**). Similarly, NRM at 3 years was comparable between patients in the high BMI group (21.4%, 95% CI, 11.70%-33.03%) and those in the low BMI group (29.29%, 95% CI, 19.47%-39.80%; p = 0.281; **Figure 1D**). However, patients in the low BMI group had significantly higher 1-year NRM compared to those in the high BMI group ([22.01%, 95% CI,13.72%-31.55%] vs. [5.08%, 95% CI,1.32%-12.87%]; p = 0.006). Multivariate analysis showed that pre-transplant BMI did not significantly influence relapse and NRM (**Table 2**).

### 3.4 Subgroup analyses

**Figure 2:**
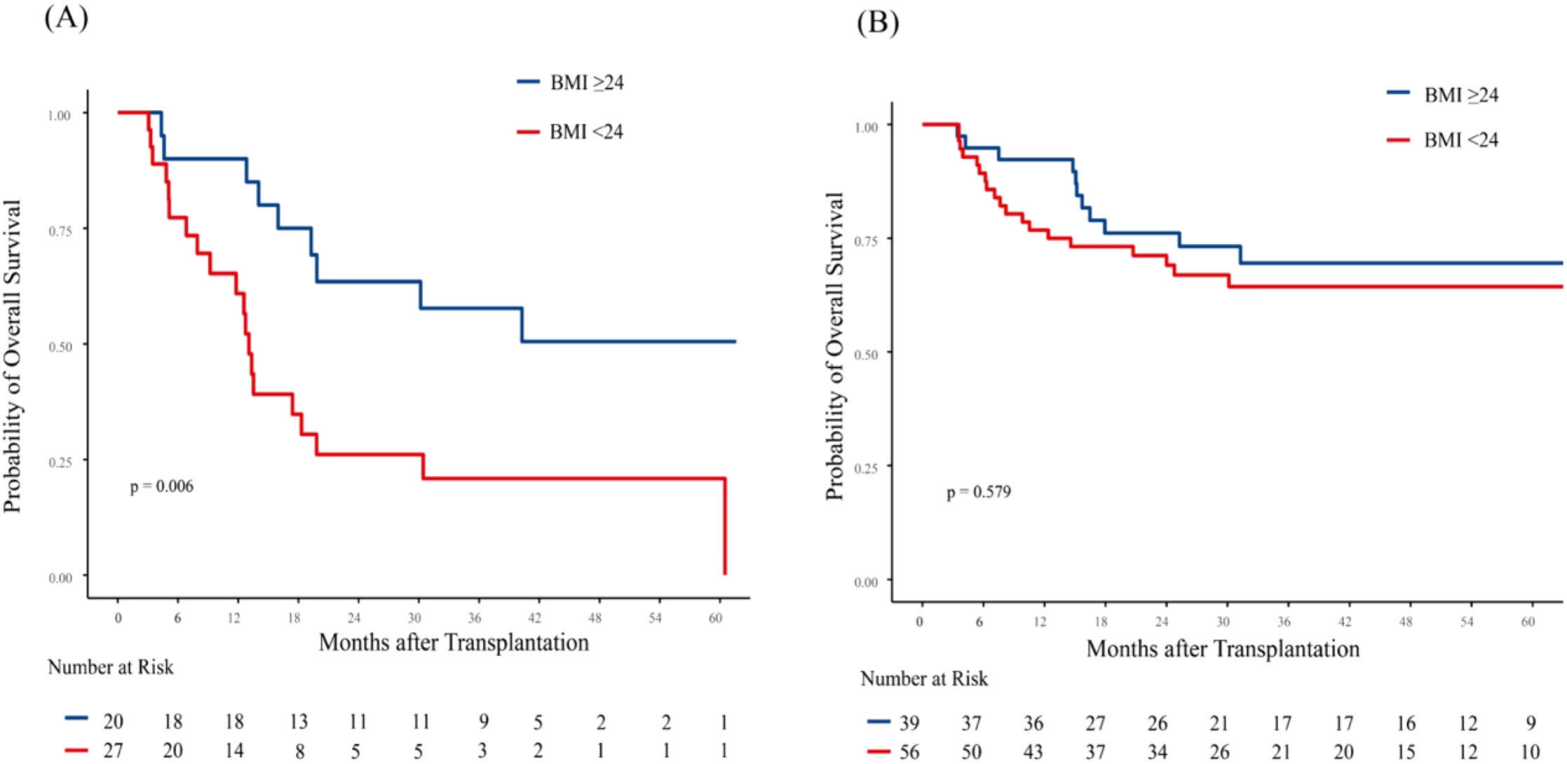
Association of body mass index (BMI) with overall survival (OS) based on disease status in AML patients. Kaplan-Meier curves for OS are shown for: **(A)** High-risk patients (NR or CR but MRD-positive), and **(B)** Low-risk patients (CR and MRD-negative). Among high-risk patients, low BMI was significantly associated with inferior OS (p = 0.006). In low-risk patients, no significant association between BMI and OS was observed (p=0.579) NR, not complete remission; CR, complete remission; MRD, minimal residual disease.

To gain a deeper understanding of the relationship between pre-transplant BMI and patient outcomes, we conducted a subgroup analysis based on disease status. Given that both CR but MRD positivity and NR status at allo-HCT significantly impacted inferior OS, we categorized these patients into high-risk group. In high-risk group, 3-year OS was 57.69% (95% CI, 39.03%-85.27%) for patients with BMI ≥ 24 kg/m² and 20.87% (95% CI, 9.28%-46.92%) for patients with BMI < 24 kg/m² (p = 0.006; **Figure 2A**). In low-risk group (CR and MRD negative), 3-year OS was 69.54% (95% CI, 55.84%-86.61%) for patients with BMI ≥ 24 kg/m² and 64.37% (95% CI, 52.53%-78.87%) for patients with BMI < 24 kg/m² (p = 0.579; **Figure 2B**). Notably, in the low-risk group, the 1-year OS was 92.31% (95% CI, 84.31%-100%) for patients with BMI ≥ 24 kg/m² and 76.79% (95% CI, 66.49%-88.68%) for those with BMI < 24 kg/m² (p = 0.054). BMI showed no significant impact on 3-year CIR and NRM in patients with different disease status at allo-HCT (**Supplementary Figure 4**).

### 3.5 Graft versus host disease

The incidence of grades II-IV aGVHD was comparable between patients in the high BMI group (22.03%, 95% CI, 12.44%-33.36%) and those in the low BMI group (30.12%, 95% CI, 20.60%-40.20%; p = 0.131). For grades III-IV aGVHD, the observed rates were 15.25% (95% CI, 7.45%-25.63%) in the high BMI group and 20.48% (95% CI, 12.55%-29.78%) in the low BMI group, respectively (p = 0.194). The cumulative incidence of GVHD-related NRM at 3 years was 9.76% (95% CI, 4.53%-17.38%) in the low BMI group and 3.39% (95% CI, 0.62%-10.49%) in the high BMI group (p = 0.154). Additionally, there was no significant difference in the proportion of steroid refractory aGVHD between the low BMI group (38.2%, 13/34) and high BMI group (33.3%, 6/18).

The rate of cGVHD at 3 years was 30.77% (95% CI, 19.39%-42.89%) for patients in the high BMI group and 34.82% (95% CI, 24.48%-45.34%) for patients in the low BMI group (p = 0.933).

## 4 Discussion

Although the impact of BMI on transplant outcomes has been extensively studied, few investigations have specifically focused on older adults, particularly in the Chinese population. In this retrospective study, we evaluated the impact of pre-transplant BMI on clinical outcomes of Chinese patients aged ≥ 50 years undergoing MAC allo-HCT. Our analysis identified low pre-transplant BMI (< 24 kg/m²) was an independent predictor of inferior OS and GRFS. These findings highlight the critical role of nutritional optimization in this vulnerable population.

A BMI ≥ 35 kg/m² has been identified as a risk factor for an increased NRM and reduced OS post-transplantation (5). However, the prevalence of obesity among Asian patients undergoing allo-HCT remains low. A study of 267 Chinese patients aged ≥ 18 years post- allo-HCT reported that 22.1% (59/267) had a BMI ≥ 25 kg/m² and 9.7% (26/267) ≤ 18.5 kg/m² by Asian standards (6). Similarly, in a large Japanese cohort of 12,050 adults receiving allo-HCT, 83.3% had BMIs in the normal-to-overweight range (WHO criteria), with only 1.9% (224/12,050) classified as obese (BMI ≥ 30 kg/m²) (15). In our cohort of 142 patients, only 3 (2.11%) had a BMI below 18.5 kg/m², and 12 (8.45%) had a BMI above 28 kg/m². We speculate this distribution likely results from two key factors: (1) lower obesity prevalence in Asian populations, and (2) exclusion of patients with extreme BMIs from MAC due to frailty or comorbidities. Thus, our findings are most relevant to elderly patients with BMIs in the normal-to-overweight range.

The impact of BMI on transplant outcomes remains controversial. While some studies associate low BMI with inferior OS, leukemia-free survival (LFS), and increased NRM in allo-HCT recipients (16, 17), others suggest that pre-HCT BMI itself does not significantly influence relapse or NRM (3). Our study provides new insights into this debate by demonstrating that pre-transplant BMI significantly affects OS and GRFS in high-risk older AML patients (≥50 years) undergoing MAC allo-HCT. Obesity has been identified as an independent risk factor for acute and extensive chronic GVHD (15). Furthermore, obesity is associated with exacerbated gut aGVHD, potentially through increased pro-inflammatory cytokine production, as demonstrated in both mouse models and patient studies (18). However, in a study analyzing elderly patients undergoing allo-HCT, it was found that obesity was not significantly associated with the incidence of aGVHD or cGVHD (9). In this study, overweight status did not elevate the risk of post-transplant GVHD in elderly patients as well. We observed that the incidence of aGVHD was higher in the low BMI group, although this trend did not reach statistical significance. Notably, patients in the low BMI group exhibited higher 1-year NRM compared to those in the high BMI group (22.01% vs. 5.08%, p=0.006). However, the mortality directly attributable to aGVHD did not significantly differ between the two groups. This lack of significance may be related to the relatively small sample size. Beyond this, we speculate that patients in the low BMI group may not have a significantly increased mortality due to a single cause, but rather exhibit a mild upward trend in multiple risk factors, such as GVHD and infections. The cumulative effect of these multifactorial risks may ultimately contribute to the observed difference in the composite endpoint.

Pretransplant disease status and MRD levels significantly impact clinical outcomes in elderly patients after allo-HCT (19, 20). Li et al. found that positive pre-transplantation MRD and active disease were both risk factors for inferior survival in AML patients after allo-HCT (21). While CIR did not differ significantly between the two groups (p = 0.411), OS was significantly lower in patients with active disease (p = 0.011). Conversely, another study reported comparable OS rates in MRD-positive AML patients or those with active disease aged 65 years or older after allo-HCT (adjusted HR = 1.033, p = 0.76) (22). In this study, we also found pre-transplantation MRD positivity or NR status was a risk factor for survival, and no significant difference in OS between the two groups. Based on this finding, we categorized patients with either MRD positivity or NR status into a single high-risk group for subsequent subgroup analysis. Subgroup analyses showed that NR or MRD-positive CR patients with BMI < 24 kg/m² had poorer OS, whereas this association was absent in MRD-negative CR patients. We believe that adequate nutritional reserves may buffer against treatment-related stressors after allo-HCT.

Global application of BMI standards shows considerable regional variability, with the World Health Organization (WHO) criteria being the most widely recognized (23). However, these universal benchmarks have been adapted by different regions to better match the specific anthropometric profiles and health data of their populations. In this study, we adopted the Chinese BMI classification instead of the Asian or WHO standard because our analysis of BMI data from 142 patients revealed that the threshold point of the HR for OS was around 24, which aligns more closely with the Chinese classification. BMI is influenced by age, and body fat percentage may vary between younger and older individuals with identical BMI (24). Besides, reliance solely on BMI for diagnosing obesity is insufficient; it is essential to incorporate additional anthropometric indicators and direct measures of body fat for a comprehensive assessment (25). Future investigations incorporating body composition analysis could provide more nuanced insights. Bioelectrical impedance analysis (BIA) can provide a comprehensive analysis of body composition and is currently the most commonly used method in clinical practice (26). The lack of additional nutritional status indicators is a limitation of this study. Future efforts will focus on integrating multiple nutritional assessment indicators to better tailor nutritional support for elderly patients after transplantation.

Our study has several limitations that should be acknowledged. First, the relatively small sample size and the single-center nature of our study limit the generalizability of our findings. Second, residual confounding cannot be ruled out despite multivariable adjustments. This includes the potential influence of trends in disease status distribution observed between BMI groups, even though these differences did not reach statistical significance. Third, our analysis focused solely on pre-transplantation BMI as the exposure variable, and did not incorporate longitudinal BMI measurements (from diagnosis to post-transplantation) to assess dynamic changes. This limits our understanding of how BMI fluctuations during the treatment course may influence outcomes. To overcome these limitations, future research should aim to include larger and more diverse patient cohorts. Multi-center studies would be particularly beneficial in providing more patients and reducing the impact of center-specific biases.

In conclusion, our study demonstrates pre-transplant BMI significantly impacts outcomes of MAC allo-HCT for older adults with AML. Greater emphasis should be placed on maintaining optimal nutritional status and providing nutritional support for these individuals, especially for high-risk patients.

## Data Availability

The original contributions presented in the study are included in the article/**Supplementary Material**. Further inquiries can be directed to the corresponding authors.
