# Supplementary material for: Impact of pre-transplant body mass index on outcomes in AML patients aged ≥ 50 years after allogeneic hematopoietic cell transplantation
Source: Front Immunol. 2025 Jul 4;16:1586523. doi: 10.3389/fimmu.2025.1586523 (PMC12270860; doi:10.3389/fimmu.2025.1586523)
Supplement: Supplementary file 1 [file DataSheet1.docx]

Supplementary Material

# Supplementary Figures legends

Supplementary Figure legends

**Supplementary Figure 1:** Impact of body mass index (BMI) on mortality hazard ratio (HR).

**Supplementary Figure 2:** Distribution of body mass index (BMI) among 142 AML patients.

**Supplementary Figure 3:** The OS stratified by disease status in 142 AML patients

**Supplementary figure 4:** Impact of body mass index (BMI) on post-transplant CIR and NRM in high-risk and low-risk patients

A.CIR in NR or CR but MRD-positive high-risk patients

B.NRM in NR or CR but MRD-positive high-risk patients

C.CIR in CR and MRD-negative low-risk patients

D.NRM in CR and MRD-negative low-risk patients

Supplementary Figure 1:


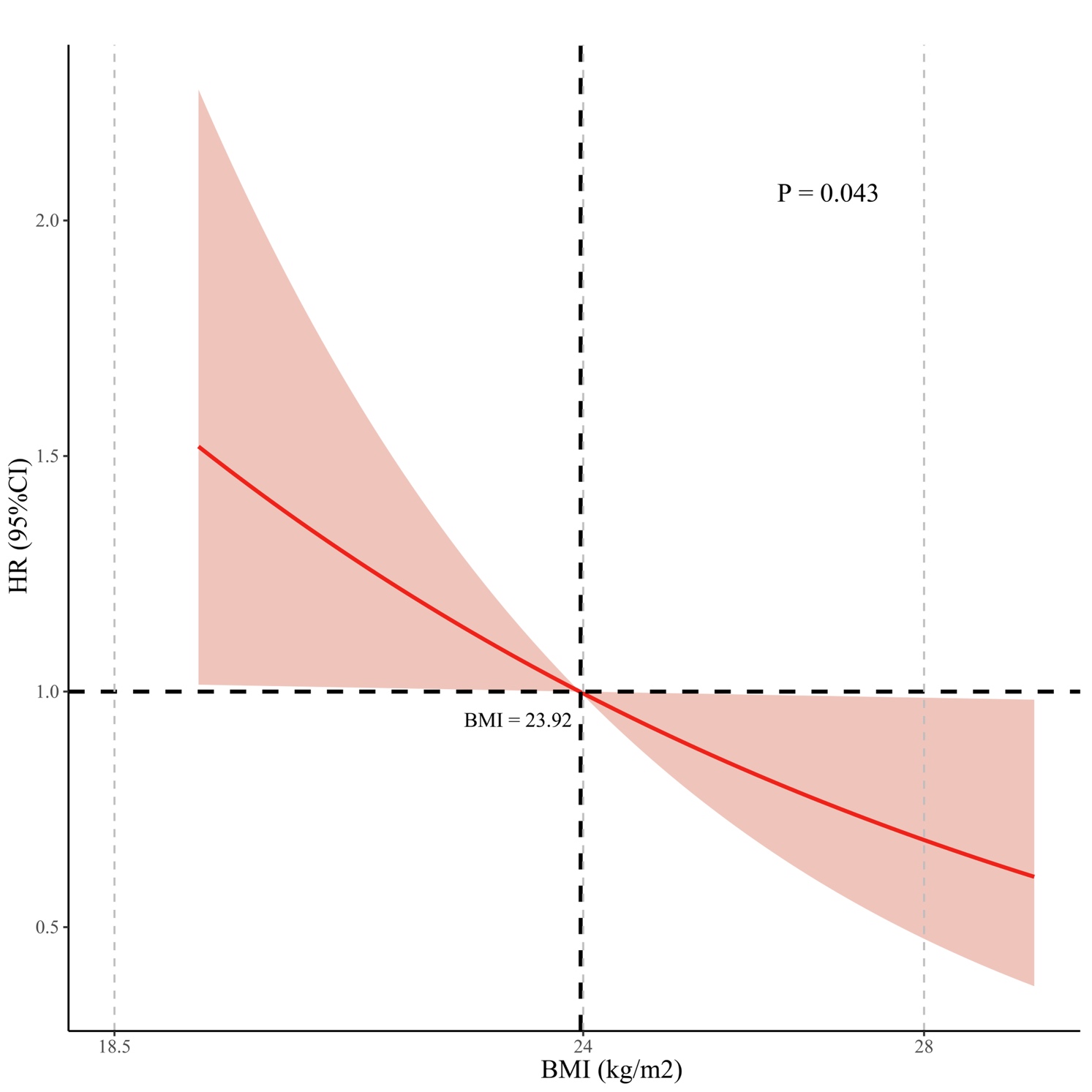


**Supplementary Fig1:**

Impact of body mass index (BMI) on mortality hazard ratio (HR). This graph explores the relationship between BMI and the HR for mortality. The red solid line represents the estimated HR. The red shaded area around the line depicts the 95% confidence interval (CI). The vertical black dashed line marks the BMI value (23.92 kg/m²) where the HR is closest to 1. Vertical dashed lines represent categories of underweight, normal-weight, overweight, and obese.

Supplementary Figure 2


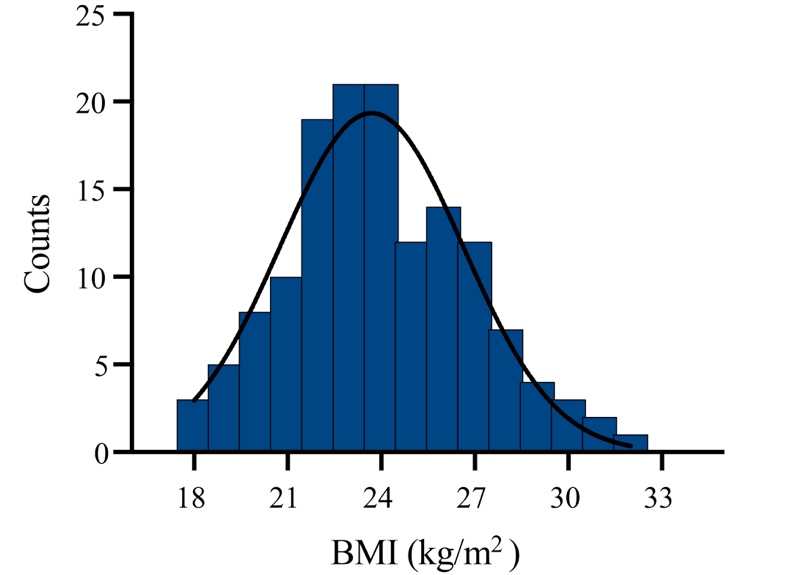


**Supplementary Fig2:** Frequency distribution histogram of body mass index (BMI) among 142 AML patients. The histogram illustrates the frequency of individuals within specific BMI ranges, showing the distribution of BMI values in the study population. The overlaid curve represents the normal distribution curve.

Supplementary Figure 3


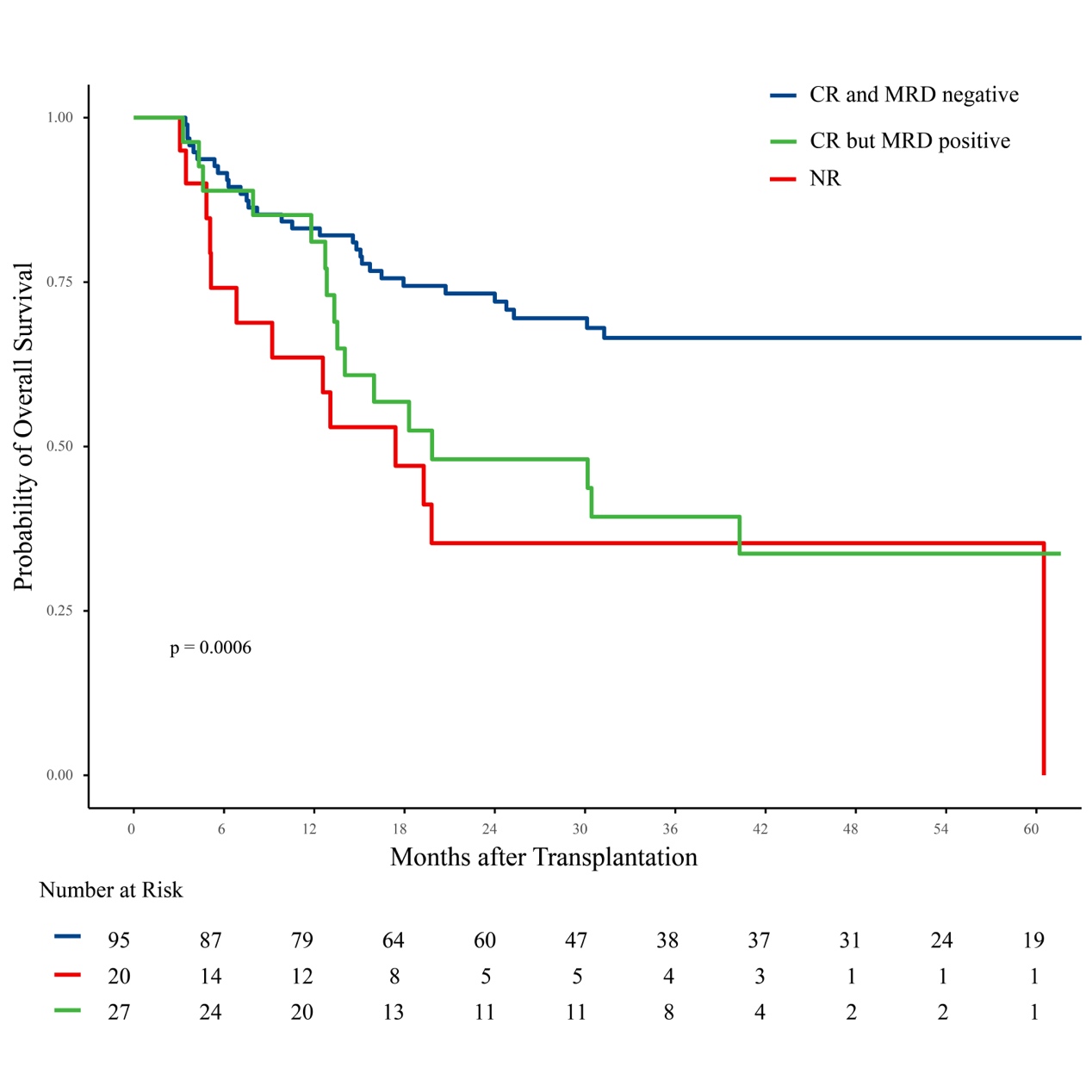


**Supplementary Figure 3:** The OS stratified by disease status in 142 AML patients

Supplementary figure 4


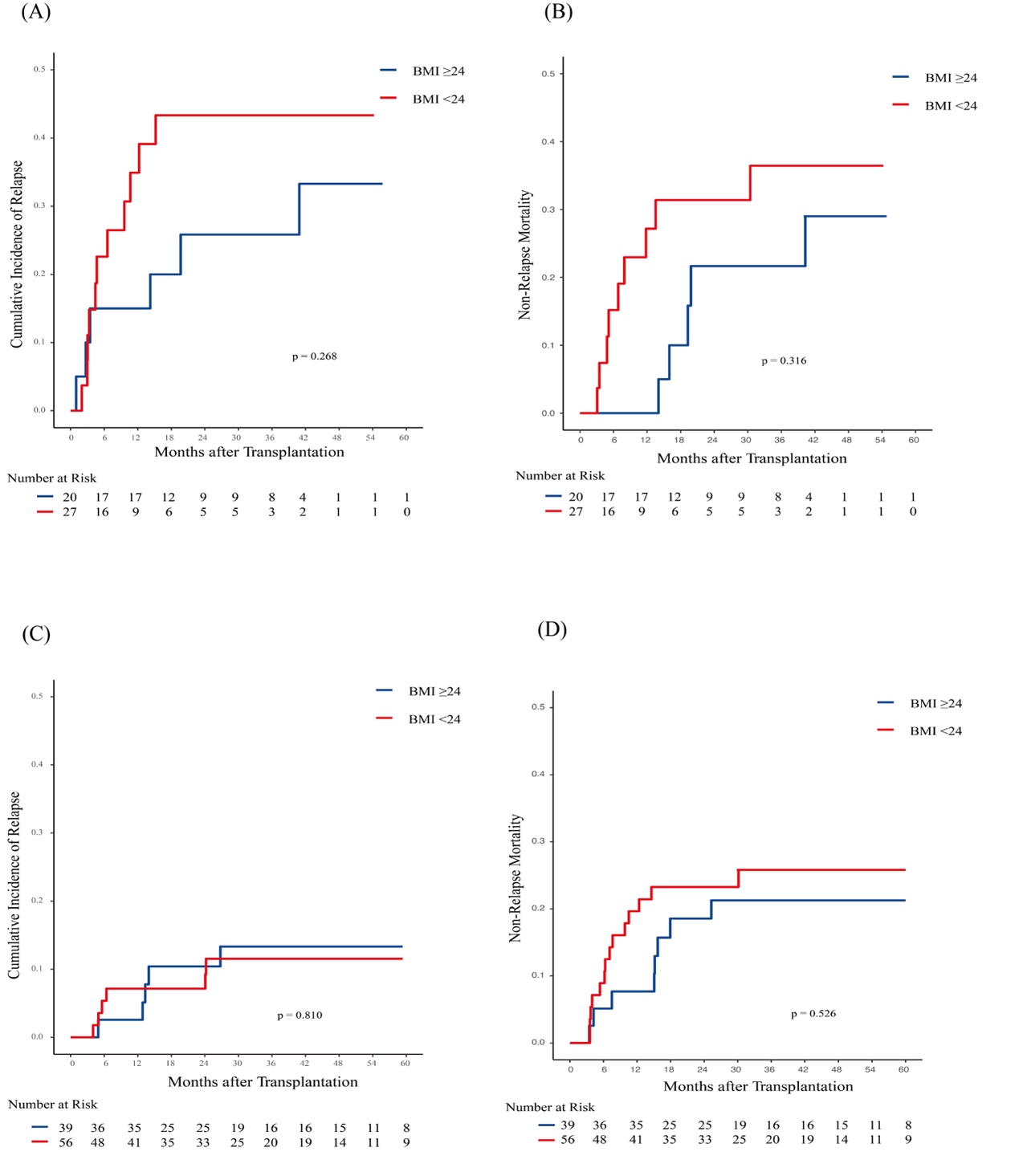


**Supplementary figure 4:** Impact of body mass index (BMI) on post-transplant CIR and NRM in high-risk and low-risk patients

A.CIR in NR or CR but MRD-positive high-risk patients

B.NRM in NR or CR but MRD-positive high-risk patients

C.CIR in CR and MRD-negative low-risk patients

D.NRM in CR and MRD-negative low-risk patients

NR: not complete remission; CR: complete remission; MRD: minimal residual disease

# Supplementary Table1: Causes of death in high-BMI and low BMI group

| Causes | High-BMI group, n (%) | | Low-BMI group, n (%) |
| --- | --- | --- | --- |
| Relapse | 9 (42.9%) | 16 (41.0%) | |
| GVHD | 2 (9.5%) | 8 (20.5%) | |
| Infection | 5 (23.8%) | 7 (17.9%) | |
| Cerebrovascular disease | 0 | 3 (7.7%) | |
| Organ failure | 1(4.8%) | 2 (5.1%) | |
| Unknown | 4 (19.0%) | 3 (7.7%) | |
| Total | 21 (100%) | 39 (100%) | |

Abbreviations: BMI, body mass index; GVHD, graft versus host disease
